# The anti-microbial peptide LL-37/CRAMP levels are associated with acute heart failure and can attenuate cardiac dysfunction in multiple preclinical models of heart failure

**DOI:** 10.7150/thno.46225

**Published:** 2020-05-15

**Authors:** Qiulian Zhou, Li-Long Pan, Ruicong Xue, Gehui Ni, Yi Duan, Yuzheng Bai, Chao Shi, Zhengnan Ren, Chengfei Wu, Guoping Li, Birgitta Agerberth, Joost PG Sluijter, Jia Sun, Junjie Xiao

**Affiliations:** 1 Cardiac Regeneration and Ageing Lab, Institute of Cardiovascular Sciences, School of Life Science, Shanghai University, Shanghai 200444, China; 2 School of Medicine, Shanghai University, Shanghai 200444, China; 3 School of Medicine, Jiangnan University, Wuxi 214122, China; 4 Department of Cardiology, the First Affiliated Hospital of Sun Yat-Sen University, Guangzhou 510080, China; 5 NHC Key Laboratory of Assisted Circulation (Sun Yat-sen University), Guangzhou 510080, PR China.; 6 Department of Cardiology, The First Affiliated Hospital of Nanjing Medical University, Nanjing 210029, China; 7 State Key Laboratory of Food Science and Technology, Jiangnan University, Wuxi 214122, China; 8 School of Food Science and Technology, Jiangnan University, Wuxi 214122, China; 9 Cardiovascular Division of the Massachusetts General Hospital and Harvard Medical School, Boston, MA 02114, USA; 10 Department of Laboratory Medicine, Division of Clinical Microbiology, Karolinska University Hospital Huddinge, F68, Stockholm, Swede; 11 Department of Cardiology, Laboratory of Experimental Cardiology, University Utrecht, University Medical Center Utrecht, 3584 CX Utrecht, The Netherlands; 12 UMC Utrecht Regenerative Medicine Center, Circulatory Health Laboratory, University Medical Center Utrecht, 3508 GA Utrecht, The Netherlands

**Keywords:** Cathelicidin, CRAMP, LL-37, Heart Failure, Serum, Biomarker, NF-κB

## Abstract

**Rationale**: Biomarkers for the diagnosis of heart failure (HF) are clinically essential. Circulating antimicrobial peptides LL-37 has emerged as a novel biomarker in cardiovascular disease, however, its relevance as a biomarker for acute HF are undetermined.

**Methods**: Acute HF patients were enrolled in this study and the serum levels of LL-37/CRAMP (cathelicidin-related antimicrobial peptide) were measured by ELISA. The receiver-operator characteristic (ROC) curve was used to determine if serum LL-37 could be a biomarker for acute HF. Mouse CRAMP (mCRAMP, mouse homolog for human LL-37) was also determined in both heart and serum samples of, transverse aortic constriction (TAC)- and isoproterenol (ISO)-induced HF mice models, and phenylephrine (PE) and angiotensin II (AngII)-induced neonatal mouse cardiomyocytes (NMCMs) hypertrophic models, both intracellular and secreted, by ELISA. The protective effects of mCRAMP were determined in TAC, ISO, and AngII-induced HF in mice while whether HF was exacerbated in AngII-infused animals were checked in mCRAMP knockout mice. The underlying mechanism for protective effects of CARMP in pathological hypertrophy was determined by using a NF-κB agonist together with rCRAMP (rat homolog for human LL-37) in AngII or PE treated neonatal rat cardiomyocytes (NRCMs).

**Results**: Serum levels of LL-37 were significantly decreased in acute HF patients (area under the curve (AUC) of 0.616), and negatively correlated with NT-proBNP. We further confirmed that mCRAMP was decreased in both heart and serum samples of TAC- and ISO-induced HF mice models. Moreover, in PE and AngII-induced NMCMs hypertrophic models, both intracellular and secreted mCRAMP levels were reduced. Functionally, mCRAMP could attenuate TAC, ISO, and AngII-induced HF in mice while CRAMP deficiency exacerbated HF. Mechanistically, the anti-hypertrophy effects of CRAMP were mediated by NF-κB signaling.

**Conclusions**: Collectively, serum LL-37 is associated with acute HF and increasing CRAMP is protective against deleterious NF-κB signaling in the rodent.

## Introduction

Heart failure (HF) is a major cause of morbidity and mortality worldwide 1,2. HF is the final stage upon different initial cardiac insults, affecting over 1% of people aged over 50 years and increases progressively with age 3. Hereby HF remains a major health epidemic, causing significant economic and public heath burdens in the world 1. Despite great advances in the treatment of HF, HF patients still have a poor prognosis, with high rates of hospital readmission and mortality 4,5. Moreover, especially for acute HF, no new therapies have improved clinical outcomes and admissions continue to increase and novel biomarkers and effective therapies are highly needed 1,6,7.

Cathelicidin-related antimicrobial peptide (CRAMP) is the rodent form of LL-37 in human 8. CRAMP can be expressed by many cells, including neutrophils, monocytes, macrophages, corneal fibroblasts, epithelial cells, B lymphocytes, T lymphocytes, NK cells, and mast cells 9,10. Besides a direct antimicrobial activity and immunomodulation effects, CRAMP has many other functional roles, including would healing, chemoattraction, cell proliferation, cell migration, apoptosis, epithelial-mesenchymal-transition, angiogenesis, and inflammation 8,11-14. Changes of CRAMP levels have been linked to cancer and a variety of autoimmune diseases, including systemic lupus erythematous, arthritis, pancreatitis, and atherosclerosis 8,15-20. We also reported that CRAMP, produced by pancreatic ß-cells, could attenuate autoimmune-induced diabetes by affecting regulatory immune cells in the pancreas 21. In the cardiovascular system, CRAMP has been found to be able to protect against cardiac fibrosis in diabetic mice 22. Besides, we and other groups have reported that CRAMP could protect against myocardial ischemia/reperfusion injury and enhance bone marrow cell retention in the treatment of cardiac dysfunction induced by myocardial infarction 9,23. Recently, CRAMP was shown to suppress cardiac hypertrophy upon induction via pressure overload 24. However, it is unclear if CRAMP can be used as a treatment for different causes of HF. Besides, circulating CRAMP has emerged as a biomarker of different cardiovascular disease 9,25, but the functional role of circulating CRAMP in HF remains to be delineated.

In the present study, we investigated if circulating LL-37/CRAMP could be a biomarker for acute HF and determined whether it can be used as a therapy for different HF backgrounds. We observed that serum LL-37 levels were significantly decreased in acute HF patients and were negatively correlated with NT-proBNP. In addition, we found that CRAMP was decreased in both heart and serum samples in transverse aortic constriction (TAC) and isoproterenol (ISO)-induced rodent HF models, and in neonatal mouse cardiomyocytes (NMCMs) cell hypertrophy models induced by phenylephrine (PE) and angiotensin II (AngII). Most importantly, CRAMP could attenuate multiple mice models of HF. In *vitro*, we proved evidence that the inhibition of NF-κB signaling contributed to the anti-hypertrophy effects of CRAMP. These findings suggest that serum CRAMP not only have a potential as a biomarker of acute HF, but also that increasing CRAMP is protective for HF induced by multiple types of cardiac stresses.

## Materials and Methods

### Acute heart failure patients

The study was approved by the Institutional Review Committee (IRC) of the First Affiliated Hospital of Sun Yat-sen University. The human investigations in this project conformed to the principles outlined in the Declaration of Helsinki. The acute heart failure (HF) patients and healthy controls were enrolled in this study from the First Affiliated Hospital of Sun Yat-sen University. The criteria for inclusion of acute HF patients were of either gender, aged over 18, with new-onset acute HF or acute decompensation of chronic HF. The diagnosis of acute HF was made by two cardiologists according to the Heart Failure Guideline of the Chinese Society of Cardiology, which was based on these facts: (i) symptoms (dyspnea at rest, dyspnea with minimal activity or peripheral edema); (ii) signs (tachypnea, pulmonary congestion with rales or elevated jugular pressure); and (iii) objective measures (chest x-ray with pulmonary congestion or NT-proBNP ≥ 1,000 pg/mL). The criteria for exclusion of acute HF patients were pulmonary embolism, serve infection or liver dysfunction, malignant carcinoma or a history of recent cardiopulmonary resuscitation. The controls were healthy volunteers who self-reported no cardiovascular diseases, had no past history of HF as well as no symptoms or signs of acute HF. The blood was collected right after the patients diagnosed. Within 1 h of venous blood collection, serum was collected by centrifugation of clotted blood at 3000 rpm for 10 min. All serum was kept in -80 ℃ refrigerator until LL-37 levels were measured by ELISA (CUSABIO).

### Animals and CRAMP knockout mice

Male C57BL/6 mice at 10-12 weeks old were purchased from Cavens' Lab Animal (Changzhou, China). The non-conditional CRAMP knockout mice were used, as we previously reported 9. All mice were bred and maintained in a specific pathogen-free (SPF) laboratory animal facility of Shanghai University (Shanghai, China). All animal experiments were conducted under the guidelines approved by the committee on the Ethics of Animal Experiments of Shanghai University.

### Cardiac hypertrophy model and CRAMP treatment

Three different cardiac pathological hypertrophy mice models were established, including TAC surgery, and Ang Ⅱ- and isoproterenol (ISO) infusion.

TAC surgery was performed as described previously 26. In brief, under sterile conditions, a 27-gauge needle placed parallel to the transverse aorta was tied by7-0 silk suture. After the needle was removed, the transverse aorta was tied between the innominate artery and left common carotid artery. Sham-operated (sham) mice were treated with the same surgery without tying the transverse aorta.

Ang Ⅱ (1.3 mg/kg/day, Sigma) was infused daily using Alzet osmotic mini-pumps (Model 2004) for 4 weeks. The control mice were infused with saline containing 0.006% acetic acid for 4 weeks.

Isoproterenol (ISO, 30 mg/kg/day, Sigma) was infused using Alzet osmotic mini-pumps (Model 2002) for 2 weeks. The control mice were infused with saline containing 0.002% ascorbic acid for 2 weeks.

Echocardiography and histological analysis were carried out 4 weeks after TAC surgery or AngII-infusion, or 2 weeks after ISO-infusion.

### Primary cardiomyocyte isolation, culture, and treatment

A change in CRAMP levels was measured in neonatal mouse cardiomyocytes (NMCMs) while functional and mechanism experiments were performed in neonatal rat cardiomyocytes (NRCMs). NMCMs and NRCMs were isolated and cultured, as previously reported 9. Cardiomyocyte hypertrophy was induced by Ang Ⅱ or PE treatment. After starvation with serum-free DMEM (Gibco, USA) for 6-8 h, cardiomyocytes were treated with Ang Ⅱ (1 μM) or PE (100 μM) for 48 h. Cardiomyocytes were simultaneously treated with CRAMP (0.1 mg/L, Innovagen AB) for 48 h. Betulinic acid (BA, Tocris), an agonist of NF-κB was used and NRCMs were treated with rCRAMP (rat homolog for human LL-37) in the presence or absence of Betulinic acid (BA, 20 μM, Tocris), for 48 h.

### ELISA

The levels of mouse CRAMP (mCRAMP, mouse homolog for human LL-37) and human LL-37 peptides were measured by ELISA (CUSABIO), according to the manufacturer's instructions. Sample candidates covered human serum, mice serum, mice heart tissue, NMCM cells and cell supernatant. Human serum were diluted at 1:200 in sample diluent. Mice serum was collected by centrifugation of clotted blood at 3000 rpm for 10 min after the blood had coagulated. Mice heart tissue was frozen in liquid nitrogen. Mice serum were diluted at 1:500 in sample diluent, whereas mice tissues were diluted at 1:100 in sample diluent. The culture supernatant and cells were collected 48 h after NMCMs were treated with Ang Ⅱ (1 μM) or PE (100 μM). Cells were collected by RIPA lysis buffer, and then diluted at 1:10 in sample diluent. At the end of the experiment, cell supernatant was collected by centrifugation of culture medium at 3000 rpm for 10 min, and CRAMP peptide levels measured by ELISA without dilutant.

### Immunofluorescent

At the end of the experiment, cell supernatant was removed and NRCMs washed in PBS and fixed in 4% paraformaldehyde for 30 min at room temperature. After washing with PBS, NRCMs were permeabilized with 0.5% Triton in PBS for 15 min. After blocking with 5% bovine serum album, NRCMs were incubated with mouse anti-α-actinin (1:200 dilution, Sigma) at 4 °C overnight. After washing with PBS, NRCMs were incubated with Fluorescein (FITC) AffiniPure Goat Anti-Mouse IgG (H+L) (Jackson) for 2 h at room temperature. Nuclei were counterstained with DAPI. After washing with PBS, 20-30 fields per sample were viewed. The size of cardiomyocytes was calculated to determine the level of hypertrophy, as induced by Ang Ⅱ or PE.

### Histological analysis

For wheat germ agglutinin (WGA) staining, frozen mouse heart sections were fixed with 4% paraformaldehyde for 15 min at room temperature. After washing with PBS, sections were incubated with WGA-FITC (1:100, Sigma) for 20 min at room temperature. After washing with PBS, nuclei were counterstained with DAPI. Finally, 40 fields per section (400x magnification) were viewed. Cell size was measured with Image J.

For hematoxylin-eosin (HE) staining, Masson's Trichrome staining and Sirius Red staining, mouse heart tissue fixed with 4% paraformaldehyde were embedded in paraffin. Then paraffin sections of heart tissue were treated by HE, Masson's Trichrome staining or Sirius Red staining. For HE staining, 40 fields per section (400x magnification) were viewed under microscope. For Masson's Trichrome staining or Sirius Red staining, 20 fields per section (200x magnification) were viewed under microscope. Then cell size and fibrosis were measured with Image J.

### Western blotting

Cardiomyocytes and mouse heart tissue were lysed as previously reported 9. After quantities by BCA assay, equal total proteins were separated in SDS-PAGE gels (10-12%). After transferred onto PVDF membranes, protein bands were blocked with 5% BSA. After washing with TBST, membranes were blotted with primary antibodies at 4°C overnight as follows: Bax (Abclonal), BCl_2_ (Affbiotech), Caspase-3 (Abclonal), Collagen I (Bioworld), Phospho-NF-κB p65 (Ser536) (Cell Signaling Technology,), NF-κB (Cell Signaling Technology), GAPDH (Bioword), and ß-actin (Bioword). Quantifications of Western Blots was done using Image J.

### Quantitative real-time polymerase chain reaction (qRT-PCR)

Total RNAs in cells or tissues were isolated with Trizol reagent (TaKaRa) and were reversely transcribed into cDNAs with RevertAid First Stand cDNA Synthesis Kit (Thermo scientific). Real-Time PCR was performed using TB Green ® Premix Ex Taq™ (TaKaRa) on Roche PCR System (LightCycler480).18S or GAPDH were used as internal controls. Primer sequences (forward and reverse) used in the present study were as follows: Rat-ANP, GAGCAAATCCCGTATACAGTGC and ATCTTCTACCGGCATCTTCTCC; Rat-BNP, GCTGCTGGAGCTGATAAGAGAA and GTTCTTTTGTAGGGCCTTGGTC; Rat-GADPH, AAGCTCACTGGCATGGCC TT and CGGC ATGTCAGATCCACAAC; Mouse-ANP, AGGCAGTCGATTCTGCTT and CGTGATAGATGAAGGCAGGAAG; Mouse-BNP, TAGCCAGTCTCCAGAGCAATTC and TTGGTCCTTCAAGAGCTGTCTC; Mouse-18S, TCAAGAACGAAAGTCGG AGG and GGACATCTAAGGGCATCAC.

### Statistical analysis

All experimental data were analyzed with SPSS 20.0 and plotted with GraphPad Prism 7.0. The statistical results were presented as mean ± SD. An independent sample T test was used for comparison between the two groups, and one-way ANOVA test was used for comparison among the three groups and above followed by Bonferroni's post hoc test. Correlation analysis was analyzed using the Spearman's method. ROC curves were assessed the sensitivity and specificity of LL-37 in predicting acute HF. P value less than 0.05 was considered statistically significant.

## Results

### The serum level of LL-37 is decreased in patients with acute HF

**Figure 1:**
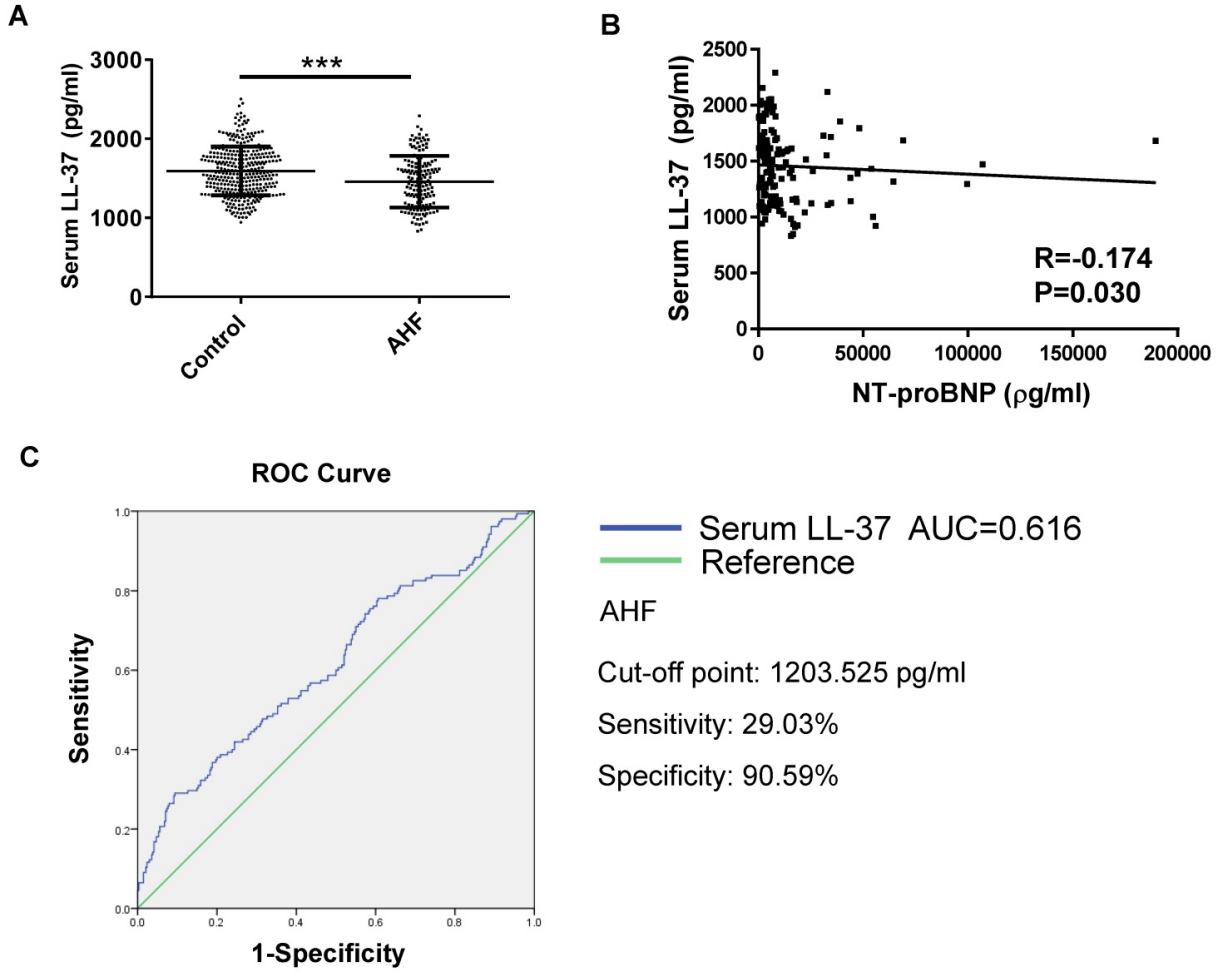
Serum levels of LL-37 in human acute heart failure (acute HF). A) The human cathelicidin peptide LL-37 was significantly decreased in human acute HF as compared to healthy controls (n=340 in healthy controls, 155 in acute HF). B) The level of LL-37 was negatively correlated with NT-proBNP. C) The receiver-operator characteristic (ROC) curve analysis demonstrated that serum LL-37 could be a biomarker for acute HF (n=340 healthy controls, 155 acute HF patients). AHF, acute heart failure. ***, P<0.001.

A total of 155 acute HF patients and 340 age- and gender- matched healthy controls were enrolled in this study. The clinical characteristics of these acute HF patients were presented in Table 1. The serum levels of human cathelicidin peptide LL-37 (human analogue of CRAMP) was measured using ELISA in acute HF patients and healthy controls. Serum LL-37 levels were significantly decreased in acute HF (Figure 1A). In addition, LL-37 levels correlated negatively with NT-proBNP (R=-0.174, P=0.030) (Figure 1B). The receiver-operator characteristic (ROC) curve suggested that serum LL-37 might be a biomarker for acute HF with an area under the curve (AUC) of 0.616 (P<0.001). Using the cutoff point of 1203.525 pg/ml, serum LL-37 predicted AHF with a specificity of 90.59% and a sensitivity of 29.03% (Figure 1C). Thus, serum LL-37 could be potentially used as a biomarker for acute HF.

### The CRAMP peptide is decreased in HF

**Figure 2:**
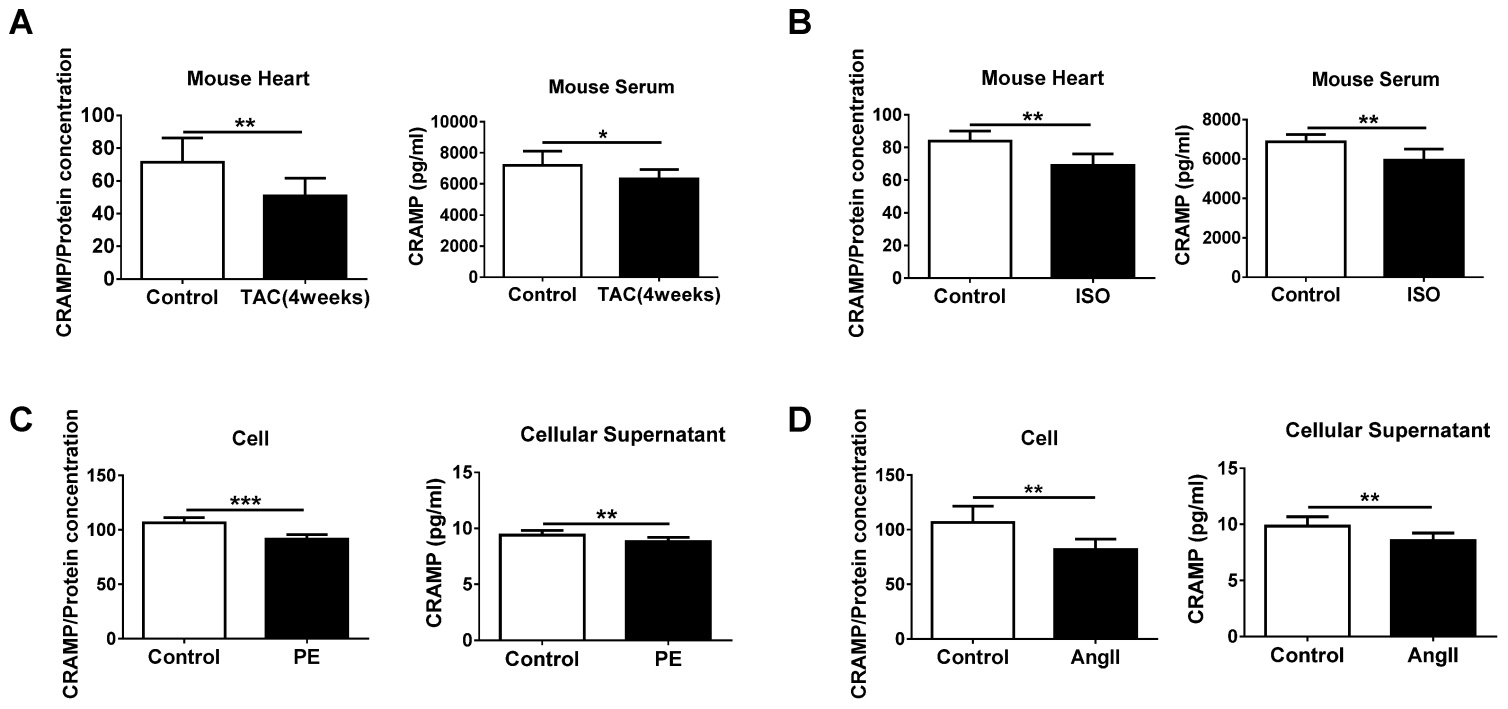
CRAMP is decreased in heart failure. A, B) mCRAMP is reduced in rodent heart and serum samples of transverse aortic constriction (TAC) (A, n=7 in control and 8 in TAC) and isoproterenol (ISO) (B, n=6 in control and 8 in ISO)-induced HF models. C, D) mCRAMP is reduced intracellularly and in cellular supernatant of neonatal mouse cardiomyocytes (NMCMs) treated with phenylephrine (PE) (C, n=9 per group) or AngII (D, n=8 per group). *, P<0.05; **, P<0.01; ***, P<0.001.

Before investigating the functional role of decreased LL-37 in acute HF, we determined the level of mCRAMP peptide in both heart and serum samples of TAC- and ISO-induced HF mice models. Consistent to the human data, the level of mCRAMP peptide was decreased in heart and serum samples of both these two models (Figure 2A, B). Furthermore, reduced intracellular and cellular supernatant levels of mCRAMP peptide in PE and AngII-treated NMCMs were also observed (Figure 2C, D). These data suggest a potential functional consequence of reduced CRAMP levels in HF and cellular hypertrophy and that increasing CRAMP might be protective for HF.

### CRAMP supplementation attenuates HF

**Figure 3:**
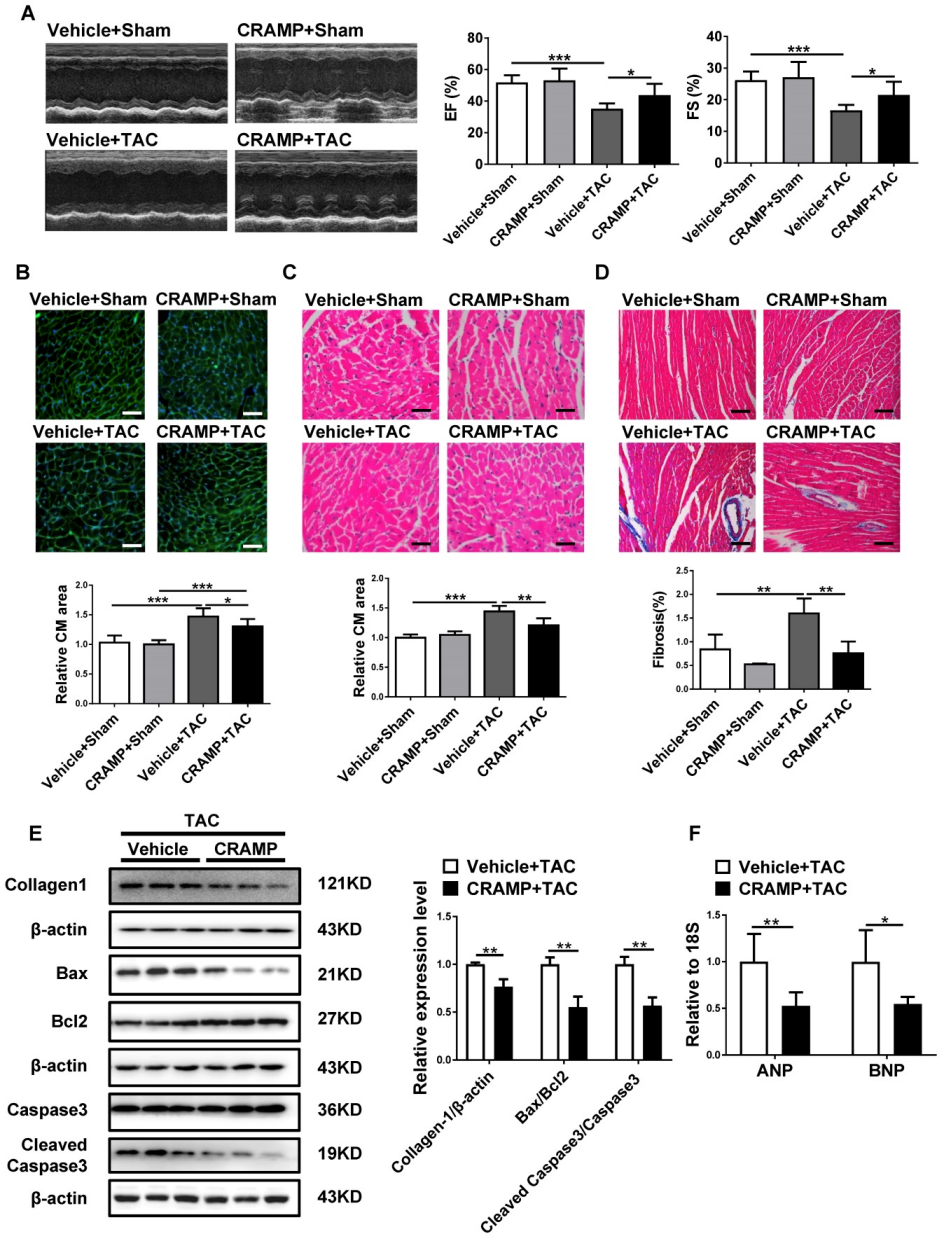
CRAMP attenuates TAC-induced heart failure. In TAC mice, mCRAMP peptide improved ejection fractions (EF) and fractional shortening (FS) (A, n=7 in Vehicle+Sham, 10 in CRAMP+Sham, 11 in Vehicle+TAC, and 9 in CRAMP+TAC), decreased cell size (B, n=7 in Vehicle+Sham, 10 in CRAMP+Sham, 11 in Vehicle+TAC, and 9 in CRAMP+TAC)(C, n=4 in Vehicle+Sham, 4 in CRAMP+Sham, 5 in Vehicle+TAC, and 5 in CRAMP+TAC), attenuated cardiac fibrosis (D, n=4 in Vehicle+Sham, 4 in CRAMP+Sham, 5 in Vehicle+TAC, and 5 in CRAMP+TAC), decreased collagen 1, the ratios of Bax/BCl_2_ and cleaved caspase 3/caspase 3 (E, n=3 per group), and reduced ANP and BNP level (F, n=6 per group). *, P<0.05; **, P<0.01; ***, P<0.001.

As CRAMP was reduced in HF, we first determined whether CRAMP could have a protective role in the heart. Of note, providing mCRAMP peptides for 2 or 4 weeks could significantly increase the level of mCRAMP peptide in the heart (Figure S1). In TAC-induced HF mice, we observed that providing the mCRAMP peptide could improve cardiac function, as demonstrated by increased ejection fractions (EF) and improved fractional shortening (FS) (Figure 3A). Moreover, WGA and HE staining both showed that the average cell size was increased in TAC mice while the mCRAMP peptide treatment could decreased this (Figure 3B, C). Moreover, interstitial cardiac fibrosis was attenuated by the mCRAMP peptide treatment in TAC mice (Figure 3D). Additionally, the expression level of Collagen 1, the ratios of Bax/BCl_2_ and cleaved caspase 3/caspase 3, and the expression level of ANP and BNP were all reduced in the TAC model upon the treatment of the mCRAMP peptide (Figure 3E, F).

**Figure 4:**
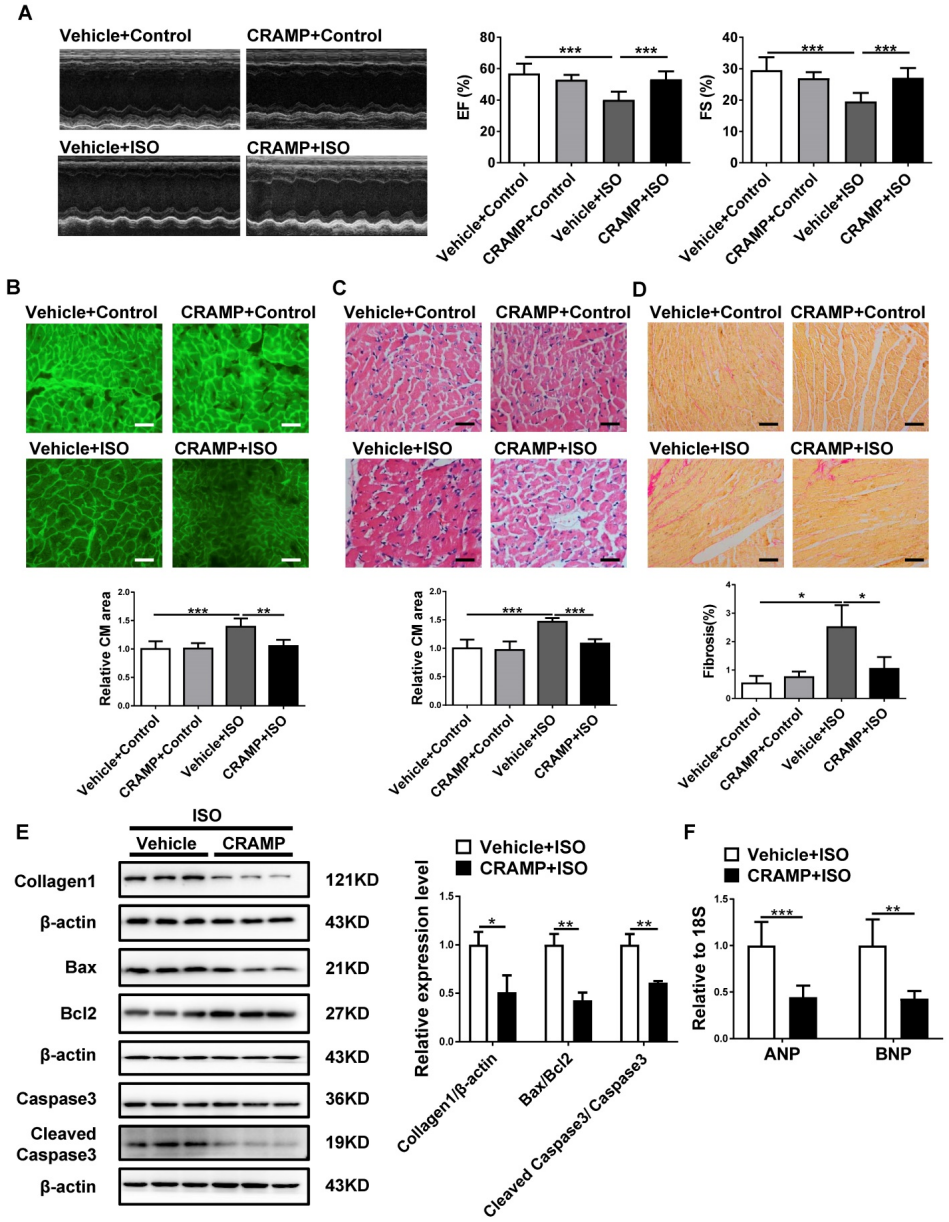
CRAMP attenuates ISO-induced heart failure. In ISO mice model, mCRAMP peptide improved EF and FS (A, n=6 in Vehicle+Control, 6 in CRAMP+Control, 10 in Vehicle+ISO, and 11 in CRAMP+ISO), decreased cell size (B, n=5 per group)(C, n=5 per group), cardiac fibrosis (D, n=5 per group), collagen 1, the ratios of Bax/BCl_2_ and cleaved caspase 3/caspase 3 (E, n=3 per group), and ANP and BNP levels (F, n=6 per group). *, P<0.05; **, P<0.01; ***, P<0.001.

**Figure 5:**
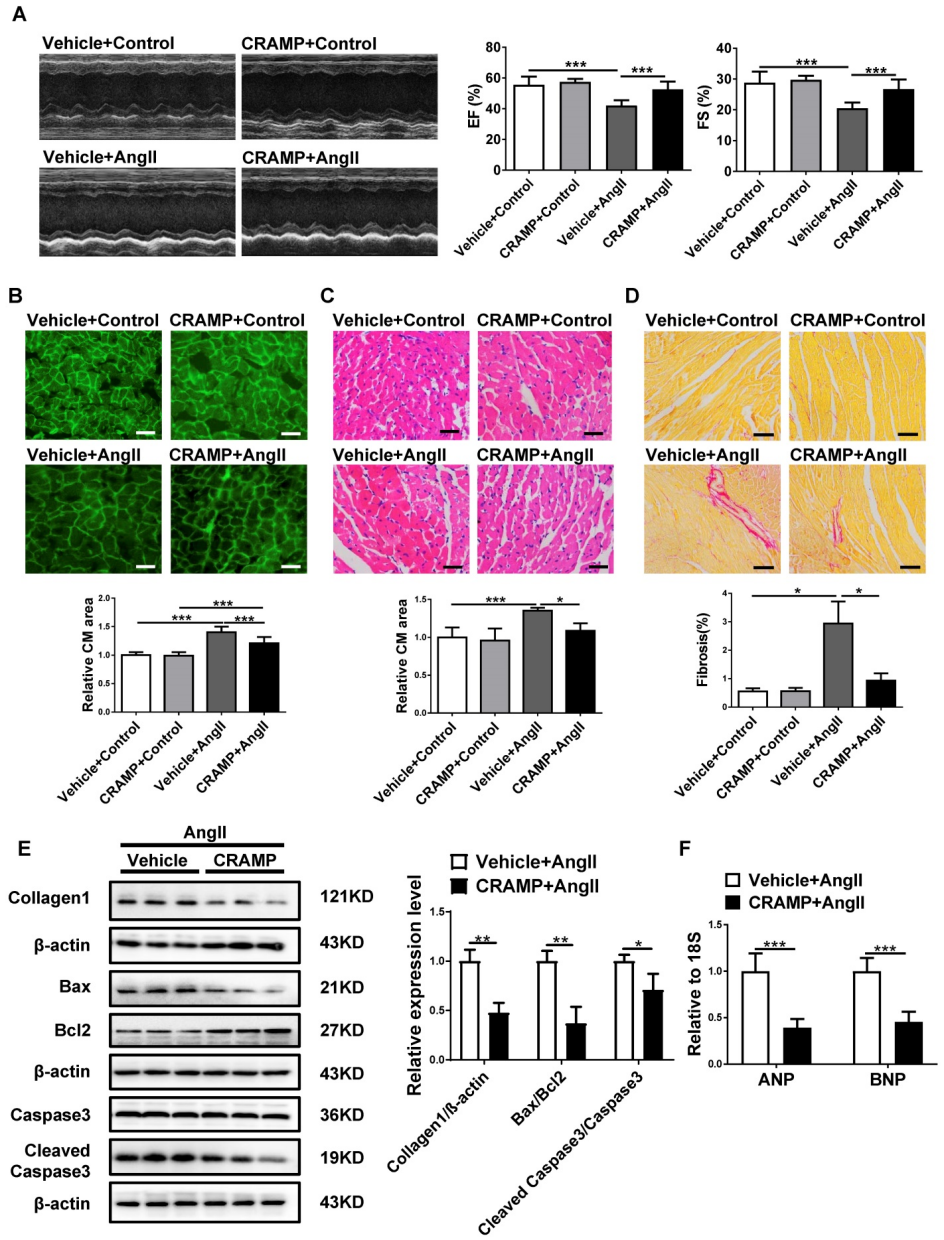
CRAMP attenuates AngII-induced heart failure. In the AngII-infused mice model, mCRAMP peptide improved EF and FS (A, n=8 in Vehicle+Control, 8 in CRAMP+Control, 8 in Vehicle+AngII, and 10 in CRAMP+AngII), decreased cell size (B, n=8 in Vehicle+Control, 7 in CRAMP+Control, 8 in Vehicle+AngII, and 7 in CRAMP+AngII)(C, n=5 in Vehicle+Control, 8 in CRAMP+Control, 5 in Vehicle+AngII, and 6 in CRAMP+AngII), cardiac fibrosis (D, n=7 in Vehicle+Control, 8 in CRAMP+Control, 4 in Vehicle+AngII, and 6 in CRAMP+AngII), collagen 1, the ratios of Bax/BCl_2_ and cleaved caspase 3/caspase 3 (E, n=3 per group), and ANP and BNP levels (F, n=6 per group). *, P<0.05; **, P<0.01; ***, P<0.001.

**Figure 6:**
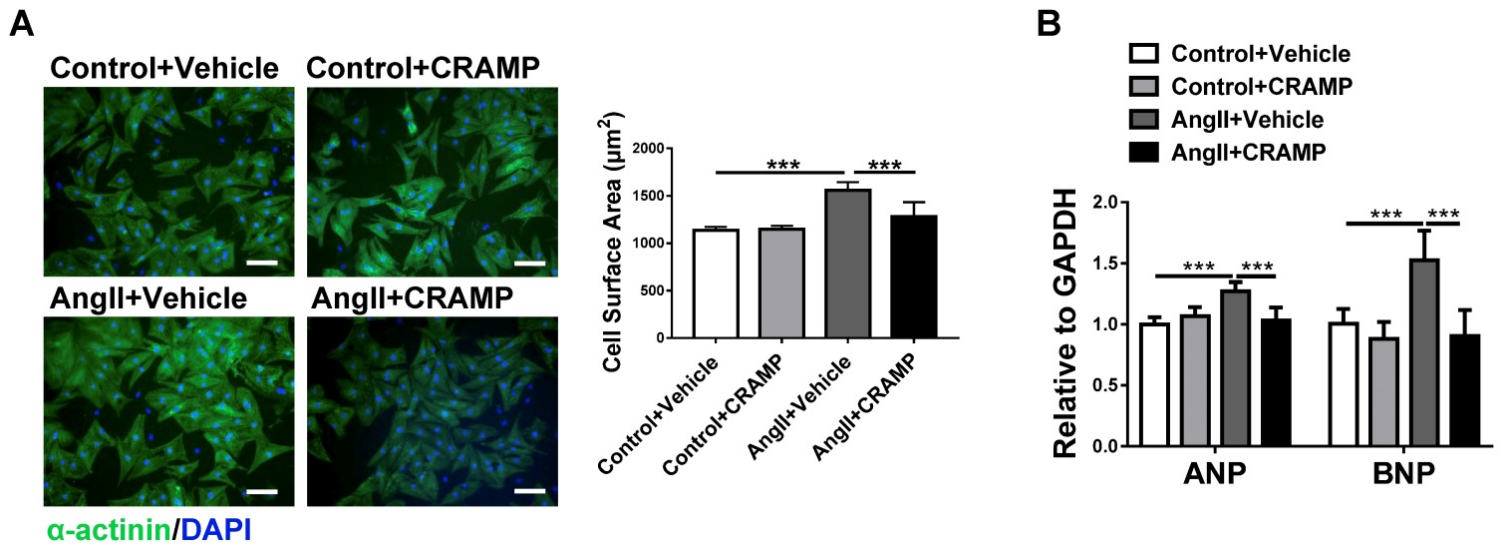
CRAMP attenuates cardiac hypertrophy in *vitro*. A, B) rCRAMP decreased cell size (A, n=6 per group) and expression levels of ANP and BNP (B, n=6 per group) in neonatal rat cardiomyocytes (NRCMs) treated with AngII. ***, P<0.001.

To determine if CRAMP can represent a common therapy for HF, we further tested the protective effects of mCRAMP peptide in other two additional HF mice models. Consistently, the mCRAMP peptide could protect from ISO- and AngII-induced HF (Figure 4, 5). Besides the *in vivo* models, we also determined the effects of CRAMP supplementation in neonatal rat cardiomyocytes (NRCMs), treated with AngII, and we observed that CRAMP could suppress pathological cardiac hypertrophy, as indicated by decreased cardiomyocytes size and mRNA expression levels of ANP and BNP (Figure 6). Collectively, increasing CRAMP levels is a common therapy for different pathological cardiac hypertrophy and HF models, both *in vivo* and in *vitro*.

### CRAMP deficiency exacerbates HF

**Figure 7:**
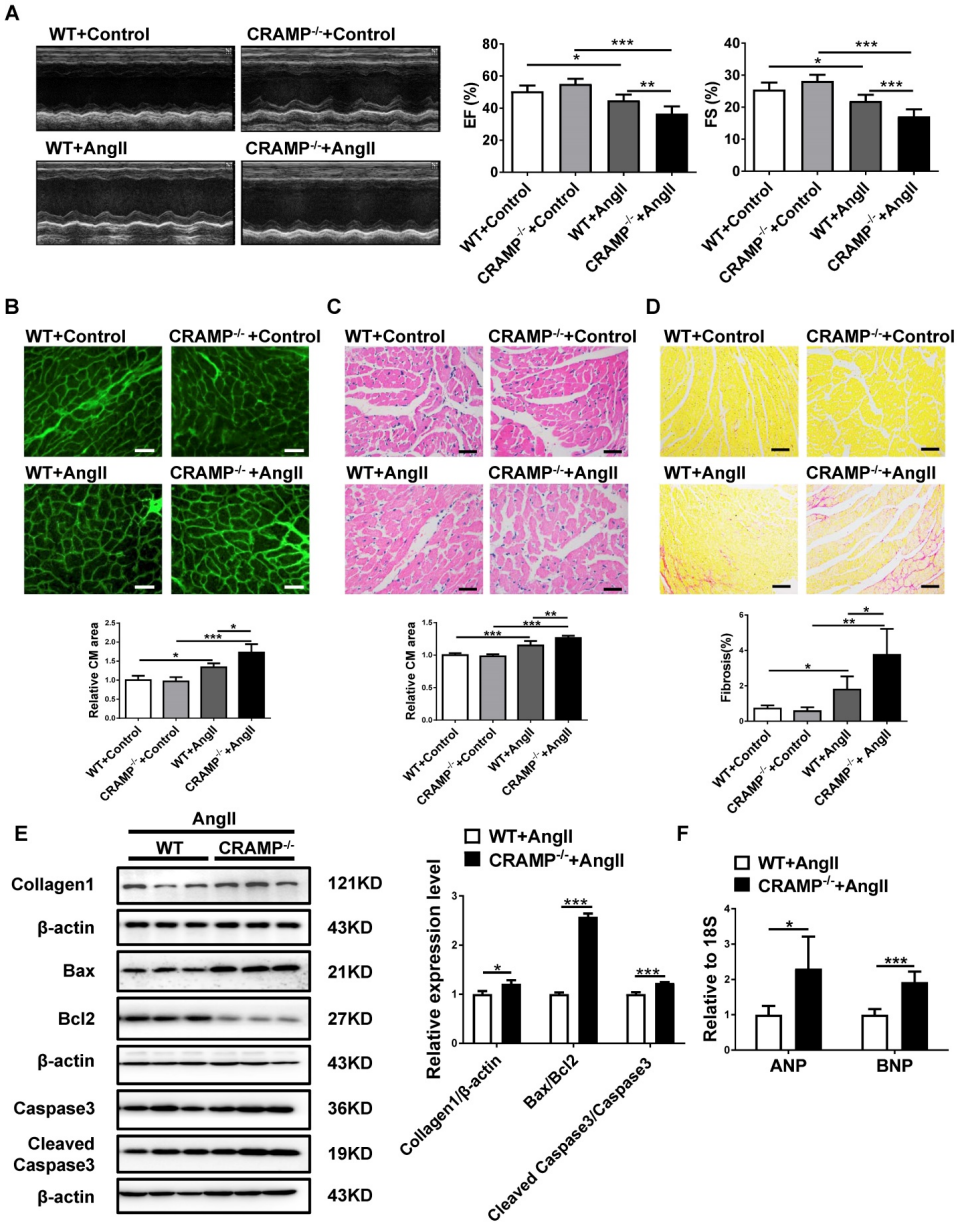
CRAMP knockout mice exacerbate AngII-induced heart failure. In AngII-infused mice, mCRAMP peptide further decreased EF and FS (A, n=8 per group), increased cell size (B, n=5 in WT+Control, 5 in CRAMP -/-+Control, 4 in WT+AngII, and 4 in CRAMP-/-+AngII )(C, n=5 in WT+Control, 6 in CRAMP -/-+Control, 6 in WT+AngII, and 6 in CRAMP-/-+AngII), cardiac fibrosis (D, n=8 per group), collagen 1, the ratios of Bax/BCl_2_ and cleaved caspase 3/caspase 3 (E, n=3 per group), and ANP and BNP levels (F, n=5 per group). *, P<0.05; **, P<0.01; ***, P<0.001.

As hypertension is the most common cause of HF 2,4,6, we also determine whether HF was exacerbated in AngII-infused animals in mCRAMP knockout mice. In mCRAMP knockout mice, cardiac function was further decreased as demonstrated by a decreased EF and FS (Figure 7A). Moreover, in the mCRAMP knockout mice, cell size, fibrosis, apoptosis and ANP and BNP expression levels were further increased in AngII mice (Figure 7B-F). Collectively, these data suggest that knockout of CRAMP gene exacerbates HF.

### The inhibition of NF-κB mediates the effects of the CRAMP peptide in suppressing pathological cardiac hypertrophy

**Figure 8:**
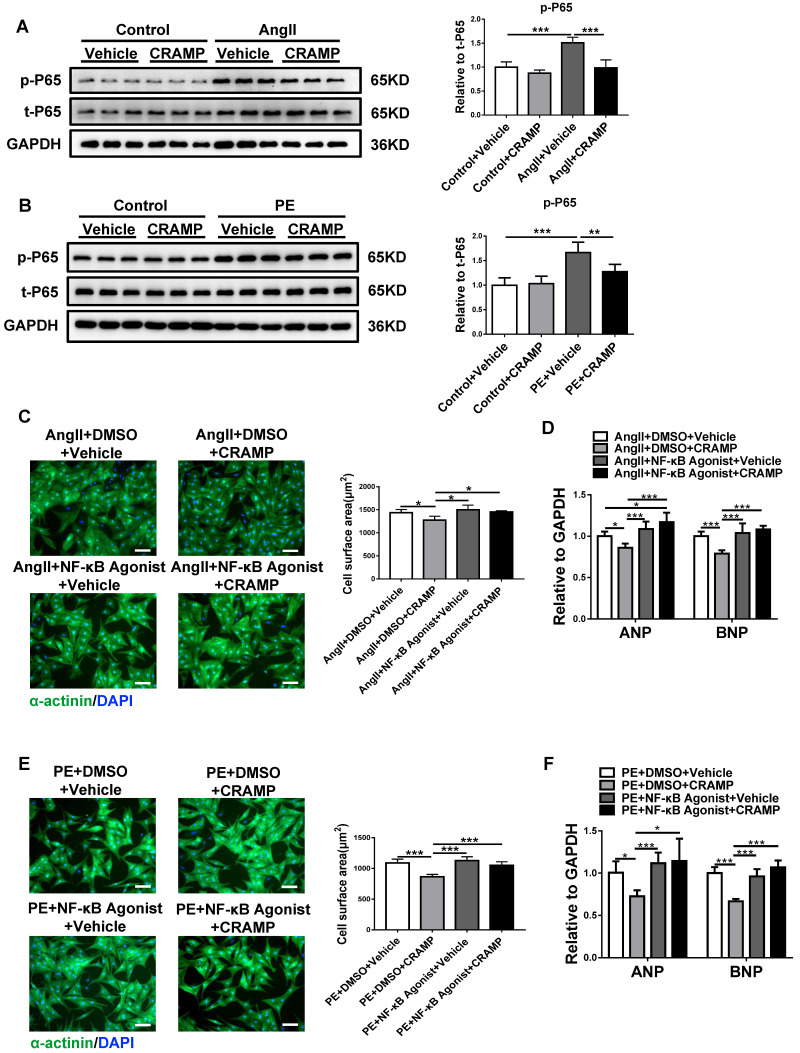
The inhibition of NF-κB contributes to the anti-hypertrophy effects of CRAMP. A, B) The p65 phosphorylation level was decreased by rCRAMP in NRCM treated with AngII (A, n=6 per group) and PE (B, n=6 per group). C, D) NF-κB agonist migrate the anti-hypertrophy effects of CRAMP in NRCM treated with AngII (C, n=6 per group) and PE (D, n=6 per group). *, P<0.05; **, P<0.01; ***, P<0.001.

To reveal the underlying mechanism for the beneficial effects of CRAMP in pathological cardiac hypertrophy, the activation of NF-κB was determined based on p65 phosphorylation levels. AngII and PE treatment in NRCMs could significantly increase phosphorylated p65, indicating the activation of NF-κB, while rCRAMP (the rat homolog for human LL-37) treatment could reduce the phosphorylation level of p65 (Figure 8A, B). To further confirm that the inhibition of NF-κB is responsible for the protective effects of the CRAMP peptide in pathological cardiac hypertrophy, NF-κB agonist was used together with rCRAMP in AngII or PE treated cardiomyocytes. We observed that NF-κB agonist abolishes the protective effects of rCRAMP in both models (Figure 8C-F). Collectively, NF-κB inhibition mediates the beneficial effects of the CRAMP peptide in pathological cardiac hypertrophy.

## Discussion

HF is a leading cause of hospitalization in people aged over 65 1, 27, 28. One in five adults currently aged 40 will develop HF in their remaining lifetime 5,7. Thus, HF is a major worldwide health epidemic, imposing significant economic and public heath burdens upon modern society 7. Nevertheless, its mortality remains extremely high with about 50% of HF patients dying within five years of diagnosis 1,29. Thus, there is an urgent need for novel biomarker and effective therapies for HF 1,29, 30. In the present study, we describe the change of circulating LL-37 as a novel biomarker for acute HF. In a cohort of 155 acute HF patients and 340 healthy controls, ROC curve analysis showed that serum LL-37 could be a biomarker for acute HF with an AUC of 0.616. Based on this initial clinical finding, we further characterized its functional role in HF. CRAMP has been emerging as a potent therapy option with a myriad of applications including myocardial ischemia/reperfusion injury, as we previously reported 9. In addition, in a pressure overload-induced cardiac hypertrophy model, CRAMP has been recently been reported to be protective 24. However, if CRAMP could be a common therapy for HF remains unclear. Here, we provide novel evidence that CRAMP can be protective for HF based on multiple models both in *vitro* and in *vivo*. More generally, this study supports an emerging paradigm that circulating CRAMP may not only serve as a reporter of disease, but may also play a functional role in the pathogenesis of human disease.

Clinically, novel biomarkers for acute HF are highly desired 31. Increasing evidence has shown that circulating LL-37 levels can change upon different stresses or injuries 32-36. Circulating LL-37 levels have been widely investigated to be increased in many infectious diseases or inflammatory conditions, including pulmonary infectious diseases, tuberculosis meningitis, and active tuberculosis 33-35,37,38. Besides, increased circulating LL-37 has also been identified in mental diseases including euthymic patients with bipolar disorder, and elderly patients with depression 32,39. The elevated serum LL-37 levels in these patients may reflect inflammatory activation 32,39. However, in systemic sclerosis (SSc) patients with interstitial lung disease (ILD), lower circulating LL-37 level has been found as compared to SSc patients without ILD and healthy persons 36. The decrease of LL-37 may lead to impairment of angiogenesis and epithelial proliferation in the lung 36. Moreover, circulating LL-37 was also decreased in acute ST-segment elevation myocardial infarction 25. In this study, we found that circulating LL-37 was significantly decreased in acute HF and could be a biomarker of acute HF. If validated in a larger independent cohort, assessment of circulating LL-37 levels in acute HF patients could constitute a major step forward in HF diagnosis and management. Of note, the specificity of LL-37 is high, which means low false positive rate and if LL-37 is decreased, it is highly possible to be acute HF. However, the sensitivity of LL-37 is low, which means it could not be used as a screening test. LL-37 should be used combined with other biomarkers with high sensitivity in the clinic. This needs to be further confirmed in a larger cohort. Besides, saliva is emerging as a promosing diagnostic fluid 40, 41. LL-37 was also found to be decreased when comparing saliva collected from NYHA class I/II HF patients with that from healthy individuals by using Sequential Window Acquisition of All Theoretical fragment ion spectra-Mass Spectrometry (SWATH-MS) 42. It remains to be determined if LL-37 in saliva could be a diagnostic biomarker for acute HF patients.

Our results support the hypothesis that some clinically useful circulating biomarkers may be functionally implicated in disease pathogenesis 9,43. Cathelicidins are pleiotropic antimicrobial peptides that have multifunctional roles in modulating innate and adaptive immune response 14,15,20. Besides, they are also implicated in numerous homeostatic processes, such as cell proliferation, cell migration, apoptosis, angiogenesis, epithelial-mesenchymal-transition, and wound healing 8,16,22. The lack of CRAMP has been linked to inflammatory bowel diseases and gastrointestinal inflammation, acute pancreatitis and ß-cell dysfunction-associated diseases 8,15,17,21. In the cardiovascular system, CRAMP has been reported to induce platelet activation and augments thrombus formation 11. Moreover, CRAMP deficiency can exacerbate septic cardiomyopathy while CRAMP can protect against cardiac fibrosis in diabetic mice heart 13,22. We previously have reported that CRAMP could protect cardiac ischemia reperfusion injury 9. Recently, CRAMP was reported to be able to protect a single model of cardiac hypertrophy (pressure overload induced) 24. In the present study, we focus on the common role of CRAMP in HF induced by a variety of initial cardiac insults. We found that mCRAMP was decreased in two HF mice models, including TAC and ISO-infusion, and in cardiomyocytes treated with PE and AngII. Importantly, we consistently demonstrated that CRAMP could be protective for HF by using multiple models including TAC, ISO and AngII-induced mouse models. Collectively, we demonstrate that CRAMP is a potential novel common therapy for HF despite the cardiac stress.

NF-κB has been reported to participate in a wide range of essential biological processes, including cell adhesion and migration, apoptosis, inflammation, hypertrophy, metabolism and during development 44-46. Furthermore, NF-κB has also been found to contribute to many diseases such as inflammatory bowel disease, septic shock, lung fibrosis, asthma and tumor 45,47. Also, NF-κB has been implicated in cardiovascular diseases including cardiac hypertrophy and HF 44. The activation of NF-κB has been found to be necessary for pathological hypertrophy in *vitro* and suppression of the activation of NF-κB by using cardiac specific expression of a mutant IκBa super-repressor mice could attenuate cardiac hypertrophy induced by AngII-and ISO-infusion, or low-grade aortic banding 48,49. Moreover, cardiomyocytes-specific IκB kinase (IKK)/NF-κB activation could lead to HF 50. However, the function of NF-κB is complex and multi-layered and thus NF-κB can have both beneficial and detrimental roles in the cardiovascular system 44,51. In certain disease settings, the NF-κB pathway is over- or under-stimulated, leading to alterations in signaling and consequently gene expression, contributing to disease pathology 51. The timing of NF-κB activation and the cellular context ultimately determines the gene expression profile that dictates the cellular outcome 51. The p65 subunit contains the transcriptional activating domains (TADs) and is the critical subunit necessary for transcriptional modulation of NF-κB 44. In the present study, we found that NF-κB was activated in NRCMs treated with AngII or PE, while rCRAMP treatment could inhibit that. This result suggests that the inhibition of NF-κB may be responsible for the protective effects of the CRAMP peptide in pathological cardiac hypertrophy. Moreover, we also found that NF-κB agonist migrated the protective effects of CRAMP in pathological cardiac hypertrophy, demonstrating that the inhibition of NF-κB mediates the anti-hypertrophy of CRAMP. However, it was previously reported that inhibition of hypertrophy-at least through attenuation of NF-κB activation-is not sufficient to block the deleterious effects of increased cardiomyocyte apoptosis in left ventricular remodeling 48. Nevertheless, our data suggest that CRAMP attenuates pathological cardiac hypertrophy by inhibition of NF-κB at least in part. Interestingly, it was previously reported that myocardial tissue from patients with HF of various etiologies shows NF-κB activation 52,53. Patients received left ventricular assist devices was also showed enhanced function with decreased NF-κB activity in heart 54, suggesting the clinical relevance of our finding in human.

## Conclusion

In summary, serum LL-37 is associated with acute HF and increasing CRAMP is protective against deleterious NF-κB signaling in the rodent.

## Figures and Tables

**Table 1:** The characteristics of acute heart failure patients

Variable	Acute heart failure
n	155
Age, y	66.08±15.63
Gender (male,%)	106(68.39)
SBP, mmHg	127.92±28.57
DBP, mmHg	75.76±16.81
Heart rate, bpm	88.37±23.02
NYHA (no.)	
Ⅰ	1(0.65)
Ⅱ	29(18.71)
Ⅲ	71(45.81)
Ⅳ	54(34.84)
Diabetes	54(34.84)
Coronary heart disease	60(38.71)
Hypertension	92(59.35)
LDL-c, mmol/L	2.70±0.98
cTnT, ng/mL	0.07(0.03-0.21)
NT-proBNP, pg/mL	6213.00(2821.00-13372.00)
LVEF, %	45.69±18.19
WBC, *10^9	8.48(6.64-11.06)

All data were presented as an absolute number (percentage), the mean (standard deviation) or the median (25^th^-75^th^ percentile). SBP, systolic blood pressure; DBP, diastolicblood pressure; NYHA, New York Heart Association; LDL-c, low-density lipoprotein cholesterol; cTnT, cardiac troponin T; NT-proBNP, N-terminal pro-B-type natriuretic peptide; LVEF, left ventricularejection fraction; WBC, white blood cell.
