# Supplementary material for: The anti-microbial peptide LL-37/CRAMP levels are associated with acute heart failure and can attenuate cardiac dysfunction in multiple preclinical models of heart failure
Source: Theranostics. 2020 May 15;10(14):6167–81. doi: 10.7150/thno.46225 (PMC7255020; doi:10.7150/thno.46225)
Supplement: Supplementary file 1 — Supplementary figures and tables. [file thnov10p6167s1.pdf]

Supporting Information

Figure S1 CRAMP treatment increased the level of CRAMP in mice heart. mCRAMP peptide treatment for 2 weeks (A, n=6 in Vehicle, 7 in CRAMP 2W) or 4 weeks (B, n=7 per group) could significantly increase the level of CRAMP in mice heart. *, $P<0.05$; **, $P<0.01$.

Figure S1

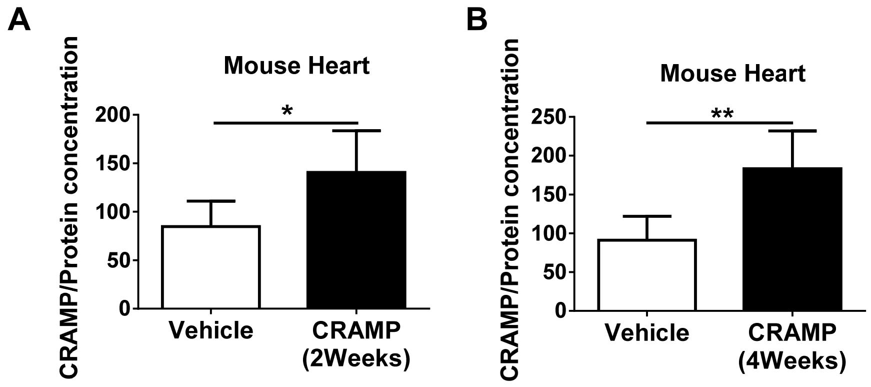

A
Mouse Heart
CRAMP/Protein concentration
200
150
100
50
0
*
Vehicle
CRAMP
(2Weeks)
B
Mouse Heart
CRAMP/Protein concentration
250
200
150
100
50
0
**
Vehicle
CRAMP
(4Weeks)
